# The androgen receptor CAG repeat polymorphism and modification of breast cancer risk in *BRCA1 *and *BRCA2 *mutation carriers

**DOI:** 10.1186/bcr971

**Published:** 2004-12-16

**Authors:** Amanda B Spurdle, Antonis C Antoniou, David L Duffy, Nirmala Pandeya, Livia Kelemen, Xiaoqing Chen, Susan Peock, Margaret R Cook, Paula L Smith, David M Purdie, Beth Newman, Gillian S Dite, Carmel Apicella, Melissa C Southey, Graham G Giles, John L Hopper, kConFaB, EMBRACE Study Collaborators, ABCFS, AJBCS, Georgia Chenevix-Trench, Douglas F Easton

**Affiliations:** 1 Queensland Institute of Medical Research, Brisbane, Australia; 2 Cancer Research UK Genetic Epidemiology Unit, University of Cambridge, Cambridge, UK; 3 School of Public Health, Queensland University of Technology, Brisbane, Australia; 4 University of Melbourne, Melbourne, Australia; 5 The Cancer Council Victoria, Carlton, Australia

**Keywords:** *AR*, *BRCA1*, *BRCA2*, modifier

## Abstract

**Introduction:**

The androgen receptor (*AR*) gene exon 1 CAG repeat polymorphism encodes a string of 9–32 glutamines. Women with germline *BRCA1 *mutations who carry at least one *AR *allele with 28 or more repeats have been reported to have an earlier age at onset of breast cancer.

**Methods:**

A total of 604 living female Australian and British *BRCA1 *and/or *BRCA2 *mutation carriers from 376 families were genotyped for the *AR *CAG repeat polymorphism. The association between *AR *genotype and disease risk was assessed using Cox regression. *AR *genotype was analyzed as a dichotomous covariate using cut-points previously reported to be associated with increased risk among *BRCA1 *mutation carriers, and as a continuous variable considering smaller allele, larger allele and average allele size.

**Results:**

There was no evidence that the *AR *CAG repeat polymorphism modified disease risk in the 376 *BRCA1 *or 219 *BRCA2 *mutation carriers screened successfully. The rate ratio associated with possession of at least one allele with 28 or more CAG repeats was 0.74 (95% confidence interval 0.42–1.29; *P *= 0.3) for *BRCA1 *carriers, and 1.12 (95% confidence interval 0.55–2.25; *P *= 0.8) for *BRCA2 *carriers.

**Conclusion:**

The *AR *exon 1 CAG repeat polymorphism does not appear to have an effect on breast cancer risk in *BRCA1 *or *BRCA2 *mutation carriers.

## Introduction

A CAG length polymorphism within exon 1 of the androgen receptor (*AR*) gene encodes a string of 9–32 glutamines. Even within this normal range, CAG repeat number is inversely associated with AR-mediated transcriptional activation *in vitro *[1,2]. Involvement of the AR in breast tumourigenesis is suggested by the existence of inactivating germline mutations in the hormone-binding domain in male breast cancer patients [3,4], and by splice variants that disrupt the transactivation domain in female breast tumours and tumour cell lines [5].

There is evidence that suggests an association between longer *AR *CAG repeat length – representative of less active AR – and breast cancer risk at the population level (for review, see Lillie and coworkers [6]). Using slightly variable definitions of shorter and longer allele size across studies, one study reported a significant twofold increased risk for breast cancer, another three studies reported a slightly increased risk for breast cancer (1.2- to 1.4-fold), and another reported a 1.7-fold increased risk limited to individuals with a first-degree family history of breast cancer [6].

Several studies have been undertaken to assess the role of the CAG repeat polymorphism as a modifier of breast cancer risk in *BRCA1 *and *BRCA2 *mutation carriers. The hypothesis-generating study [7] examined *AR *CAG length in 304 female *BRCA1 *mutation carriers (54% with breast cancer), and assessed breast cancer risk associated with CAG length as a continuous variable, and at a number of different cut-points. That study reported a 1.8-fold relative risk (95% confidence interval [CI] 1.1–3.1; *P *= 0.03) among the small subgroup of women with at least one *AR *allele of 28 or more CAG repeats in length, with relative risks of 2.6 (95% CI 1.5–4.7; *P *< 0.001) and 4.5 (95% CI 1.3–15.2; *P *= 0.02) for the cut-points ≥ 29 CAG repeats and ≥ 30 CAG repeats, respectively. Subsequent studies have reported conflicting results. A relative risk of 1.1 (95% CI 0.5–2.6) was observed for women with 28 or more CAG repeats from a pooled analysis of 188 *BRCA1 *and *BRCA2 *mutation carriers [8], whereas a study of 227 *BRCA1 *and *BRCA2 *mutation carriers reported shorter mean CAG repeat number in women with breast cancer diagnosed before age 42 years as compared with those diagnosed after this age [9].

It has been hypothesized that *AR *alleles with decreased transactivation might act directly to result in decreased breast cell proliferation, or possibly indirectly via an endocrine or paracrine mechanism whereby altered levels of circulating or stromal hormones might affect mammary epithelial growth [7]. Subsequent biochemical studies reported a protein–protein interaction between the AR protein and particular regions of both the BRCA2 and BRCA1 proteins. BRCA2 has been reported to enhance androgen-dependent AR activity [10]. Although that study also reported that an expressed truncated BRCA2 protein encoded by the *BRCA2 *L1042X mutation failed to enhance AR transactivation [10], it did not examine the effect of *BRCA2 *mutation position in general, or which domains of BRCA2 are required for the BRCA2–AR interaction.

BRCA1 has also been reported to enhance androgen-dependent AR transactivation [11,12]. Mammalian two-hybrid assays have shown that BRCA1 amino acids 231–1314 and 1560–1863 are responsible for this direct interaction, with BRCA1 region 758–1064 observed to bind specifically to the AR amino-terminal domain containing the glutamine repeat [11]. Androgen response assays indicated that, although *BRCA1 *mutations across the gene all reduce AR activity enhancement as compared with the wild-type, the effect was more marked for mutations up to amino acid 1365 [11]. This was particularly true for a mutation that caused truncation at amino acid 772 within the BRCA1–AR amino-terminus interaction domain, which had 20% activity relative to the wild-type BRCA1. The effect of the *AR *CAG repeat length on BRCA1–AR interactions has not yet been investigated. However, molecular studies provide some support for a biochemical interaction between *AR *CAG repeat length and *BRCA1 *mutation status, in that the decreased AR transactivation observed *in vitro *with increasing glutamine length was only observed in the absence of coexpressed BRCA1 [12].

These data suggest that an association of *AR *CAG repeat length with increased breast cancer risk may be found only in *BRCA1 *or *BRCA2 *mutation carriers (and not individual germline without *BRCA1 *or *BRCA2 *mutations), and that *AR*-dependent modification of cancer risk in *BRCA1 *and *BRCA2 *mutation carriers may differ according to which gene is mutated, and the mutation position relative to AR-binding site. To evaluate further the evidence for an association between *AR *CAG repeat length and breast cancer risk in *BRCA1 *and *BRCA2 *mutation carriers, we genotyped the polymorphism in a large series of female mutation carriers.

## Methods

### Subjects

The distribution of samples according to source, gene and cancer status is shown in Table 1.

A total of 604 living female Australian and British carriers of pathogenic *BRCA1 *or *BRCA2 *mutations were identified in 376 families from the following sources: the Epidemiological study of *BRCA1 *and *BRCA2 *Mutation Carriers (EMBRACE; ), the Kathleen Cuningham Consortium for Research into Familial Breast Cancer (kConFaB; ), the Australian Jewish Breast Cancer Study (AJBCS) [13], and the Australian Breast Cancer Family Study (ABCFS) [14,15]. EMBRACE recruits participants from among women and men referred for genetic testing at clinical genetics centres in the UK and Eire. kConFaB recruits participants from multiple-case breast and ovarian cancer families referred for genetic testing at family cancer clinics in Australia and New Zealand. AJBCS recruits Ashkenazi Jewish women reporting a personal or family history of breast or ovarian cancer in a first- or second-degree relative, and living in Melbourne or Sydney, Australia. Finally, ABCFS is a population-based case–control-family study that includes women with a first primary breast cancer recruited through the Victorian and New South Wales cancer registries, and their affected and unaffected relatives. Apart from index cases recruited through cancer registries for the ABCFS, the cancer status of participants was based on self-report.

For samples recruited through EMBRACE, a pathogenic mutation was defined as an established disease-causing mutation under the classification scheme used by Breast Cancer Information Core . For samples recruited through kConFaB, AJBCS and ABCFS, mutations were classified as pathogenic according to the criteria established by kConfab . Specifically, the criteria specify the following as being pathogenic: all truncating mutations, unless there is clear evidence that the mutation is a single nucleotide polymorphism (e.g. terminal *BRCA2 *variant); and any variant that is well characterized in family studies of multiple generations, and not found in control individuals, that results in a nonconservative amino acid substitution, and occurs in a residue conserved across species and in a functional domain. All mutations included in the study that were shared across sites were classified as pathogenic according to both routes of definition.

Within Australia, ethical approvals were obtained from the ethics committees of the Peter MacCallum Cancer Institute, The Prince of Wales Hospital, The University of Melbourne, The Cancer Council New South Wales, The Cancer Council Victoria and the Queensland Institute of Medical Research. Ethical approval for the EMBRACE study was obtained from the Eastern Multicentre Research Ethics Committee and the relevant local ethics committees. Written informed consent was obtained from each participant.

### Molecular methods

The *AR *exon 1 CAG repeat length was measured by fluorescent polymerase chain reaction PAGE methodology, using the ABI Prism 373 Genescan (Applied Biosystems, Foster City, CA, USA) and Genotyper systems (Applied Biosystems). Details of this method were previously reported [16]. Genotyping was successful for 375 out of 382 (98%) *BRCA1 *carriers, 218 out of 221 (99%) *BRCA2 *carriers, and the single carrier of both a *BRCA1 *and a *BRCA2 *mutation.

### Statistical methods

Individuals with a first diagnosis of primary invasive breast cancer were considered to be affected, whereas individuals with no reported breast or ovarian cancer were censored at age at interview. Individuals with a first diagnosis of primary ovarian cancer were censored as unaffected at age at onset of ovarian cancer, and selected analyses were also performed in which individuals with a first primary diagnosis of ovarian cancer were excluded. All individuals were censored at age of prophylactic mastectomy. Individuals reporting prophylactic surgery included a single *BRCA2 *carrier who was subsequently diagnosed with multiple breast cancers 4 and 5 years after surgery, and an additional 12 unaffected individuals (7 *BRCA1 *and 5 *BRCA2 *carriers) with surgery 1–11 years before interview (average 3 years). Prophylactic oophorectomy of affected and unaffected individuals was controlled for by adjustment as a time-dependent covariate, as described below.

Linear regression was used to assess the association of *AR *CAG repeat length (smaller allele size, larger allele size and average allele size) with potential confounders within the subset of 364 *BRCA1 *carriers and 209 *BRCA2 *carriers for whom information was available. The potential confounders included year of birth (categorized into subgroups 1910–1949, 1950–1959 and 1960–1979), age at menarche (categorized as ≤ 11, 11.5–12, 12.5–13, 13.5–14 or ≥ 14.5 years), oral contraceptive pill use (ever/never) and parity (categorized as 0 or ≥ 1 live births before censored age). Questionnaire information was available from participants on age at first and last live birth, but not age at each live birth. Hence, it was not possible to assess association with parity as an absolute number of live births before censored age, but rather only as a never/ever variable. Associations were assessed separately for affected and unaffected women.

The primary analyses of association between *AR *genotype and disease risk were performed using Cox regression with time to breast cancer onset as the end-point. *AR *CAG repeat length was defined as follows: a binary variable, defined by cut-points investigated in the hypothesis-generating study conducted by Rebbeck and coworkers [7] (namely one or more allele of ≥ 28 CAG repeats, ≥ 29 CAG repeats, or ≥ 30 CAG repeats); or a continuous variable, using the length of the smaller of the two alleles (*AR *small CAG), the larger of the two alleles (*AR *large CAG), and the average length of a participant's two alleles (*AR *average CAG). Rate ratios (RRs) and 95% CIs were estimated with adjustment for source group (as indicated in Table 1) and ethnicity (non-Jewish Caucasian, Jewish, other). Analyses were complicated by the fact that more than one mutation carrier could come from the same family and could not therefore be considered independent. Standard Cox regression provides unbiased RR estimates but their standard errors and CIs are incorrect. This was rectified by computing the confidence limits for the RRs using Huber's sandwich estimator of the covariance matrix [17]. This allows for variation between carriers from the same family without modelling their dependence explicitly. Further analyses adjusted for oophorectomy and parity as time-dependent covariates, and for age at menarche, oral contraceptive pill use and year of birth. Oophorectomy before censored age at interview or diagnosis of breast cancer was reported by 39 *BRCA1 *carriers (10 with primary breast cancer) and 21 *BRCA2 *carriers (9 with primary breast cancer) with genotype information available.

RRs were estimated separately for *BRCA1 *and *BRCA2 *carriers, including in both analyses the single individual with a mutation in both genes. Models adjusting for only group and ethnicity included all individuals with genotype information, namely 376 *BRCA1 *carriers (with 200 events) and 219 *BRCA2 *carriers (with 122 events). The sample size for *BRCA1 *carriers with a putative risk allele was 28 (14 events) for the ≥ 28 CAG cut-point, 26 (13 events) for the ≥ 29 CAG cut-point, and 11 (4 events) for the ≥ 30 CAG cut-point. Similarly, for *BRCA2 *carriers it was 17 (10 events) for the ≥ 28 CAG cut-point, 14 (7 events) for the ≥ 29 CAG cutpoint, and 11 (5 events) for the ≥ 30 CAG cut-point. Full models adjusting for year of birth and additional hormonal variables included the 364 *BRCA1 *and 219 *BRCA2 *carriers with full information on potential confounders, comprising 193 and 116 events, respectively.

In addition, analyses were carried out separately for subgroups of *BRCA1 *and *BRCA2 *carriers defined by mutation position in relation to proposed AR-binding domains, and/or *in vitro *data regarding mutation effect on AR transactivation [10,11]. For *BRCA1*, subgroups were defined by the mutation position either relative to amino acid 1365 (< or ≥ nucleotide 4213), because mutations 5' of amino acid 1365 have been shown to have a markedly decreased effect on AR transactivation [11]. This created subgroups including 314 and 62 individuals. In addition, *BRCA1 *subgroups were defined by mutation relative to amino acid 1065 (< or ≥ nucleotide 3311), because this defines the 3' end of the *BRCA1 *fragment shown *in vitro *to bind the *AR *amino-terminal domain containing the CAG-encoded polyglutamine tract [11], creating subgroups of 210 and 166 individuals. *BRCA2 *subgroups were defined by mutation relative to amino acid 1042 (< or ≥ nucleotide 3352), because it has been shown that the *BRCA2 *L1042X mutation does not enhance AR transactivation [10]. Subgroup sample sizes were 42 and 177. For both *BRCA1 *and *BRCA2 *subgroup analyses, the 3' and 5' subgroups were termed domain 1 and domain 2, respectively. Protein truncating and splice mutations were stratified into domain 1 or domain 2 according to their nucleotide/amino acid position, whereas all missense mutations were included in domain 2 because these nontruncating mutations may act in a dominant-negative manner.

Although the primary analysis provides a valid test of the association between a genotype and disease risk, it may not provide a consistent estimate of the RR because the disease status of the individuals may have affected the likelihood of ascertainment (for the non-population-based studies). Oversampling of affected individuals is likely, as is presentation of affected carriers at later mean age than unaffected carriers. To correct for this potential bias, we also conducted secondary analyses using the weighted Cox regression approach as described by Antoniou and coworkers (unpublished data), in which individuals are weighted such that the observed breast cancer incidence rates in the study sample are consistent with established breast cancer risk estimates for *BRCA1 *and *BRCA2 *mutation carriers. Antoniou and coworkers (unpublished data) have shown that this approach gives estimates that are close to unbiased, but with some loss of power as compared with the standard unweighted approach. Weights were computed separately for *BRCA1 *and *BRCA2 *mutation carriers using the breast cancer incidence rate estimates reported in the meta-analysis conducted by Antoniou *et al*. [18]. A global set of weights was computed because the number of mutation carriers by study was too small to compute reliable study-specific weights. Moreover, Antoniou *et al*. [18] found no significant differences in the *BRCA1 *and *BRCA2 *cancer risks by country or study centre. As for unweighted analyses, confidence limits for the risk ratio were calculated using a robust variance approach to allow for the dependence among individuals.

We evaluated the power of detecting the effects reported by Rebbeck and coworkers [7] in our samples of *BRCA1 *and *BRCA2 *mutation carriers using simulations. For this purpose, we assumed the age distribution of the affected and unaffected carriers in our sample (Table 1) and simulated among them risk factors with risk ratios 1.8 and 2.6 and the frequencies for the ≥ 28 and ≥ 29 CAG cut-points observed in our sample. The data were then analyzed using unweighted Cox regression. We conducted 1000 simulations per model. More details about the simulations are available from the authors of the present report. The power of detecting risk ratios of 1.8 and 2.6 was estimated to be 51% and 92%, respectively, for the sample of *BRCA1 *mutation carriers and 28% and 78% for the sample of *BRCA2 *mutation carriers.

R version 1.9.0 (R Foundation for Statistical Computing, Vienna, Austria) was used to perform the unweighted Cox regression, and STATA version 7 (Stata Corporation, College Station, TX, USA) was used for the weighted analyses.

## Results

The *AR *CAG length ranged from 8 to 36 repeats. There was little power to assess potential confounding or risk associated with the cut-point ≥ 30 CAG repeats, because only 11 *BRCA1 *and 11 *BRCA2 *carriers had at least one allele of this size. There was no evidence for an association between *AR *CAG repeat length and year of birth, age at menarche, or parity. There was marginal evidence for a preponderance of smaller alleles among affected *BRCA2 *carriers who reported using the oral contraceptive pill (*P *= 0.03), but this association was not seen in unaffected individuals or in *BRCA1 *affected or unaffected carriers (*P *> 0.2).

The estimated rate ratios associated with *AR *CAG repeat length are given in Tables 2 and 3. Risk estimates using the weighted Cox regression approach were very similar to the unweighted estimates, and for simplicity the unweighted estimates are shown. No associations were observed for alleles ≥ 28 CAG repeats or ≥ 29 CAG repeats – cut-points previously reported to be associated with risk. Estimated RRs were close to and not significantly different from 1 (all *P *> 0.3) and were in most instances less than 1. The number of individuals with ≥ 30 CAG repeats was too small to provide reliable risk estimates, but point estimates (0.49 [*P *= 0.1] for *BRCA1*, 0.69 [*P *= 0.5] for *BRCA2*) provided no evidence for increased risk associated with these large alleles. When *AR *CAG repeat length was considered as a continuous variable, there was no association either with average repeat length or with the length of the shorter or longer allele (*P *≥ 0.2). There was little difference between the estimates adjusted only for source group and ethnicity, and those adjusted also for year of birth, and hormonal variables oophorectomy, parity, age at menarche and contraceptive pill use. Risk estimates were not markedly different when women with a first primary diagnosis of ovarian cancer were excluded, with a RR (95% CI) for the ≥ 28 CAG cut-point of 0.85 (0.49–1.47) for *BRCA1 *mutation carriers (*P *= 0.6), and 1.12 (0.0.55–2.27) for *BRCA2 *mutation carriers (*P *= 0.8).

Results using the weighted Cox regression approach indicated that RR estimates were not materially affected by possible ascertainment biases. For example, for the ≥ 28 CAG cut-point, the RR (95% CI) adjusted for group and ethnicity was 0.74 (0.33–1.66) for *BRCA1 *carriers (*P *= 0.5) and 0.94 (0.32–2.77) for *BRCA2 *carriers (*P *= 0.9). For average CAG length, the RR (95% CI) adjusted for group and ethnicity was 1.01 (0.94–1.10) for *BRCA1 *carriers (*P *= 0.8) and 0.96 (0.82–1.11) for *BRCA2 *carriers (*P *= 0.6). These results are consistent with the findings of Antoniou and coworkers (unpublished data), in which both weighted and unweighted Cox regression analyses give similar estimates when the true RR is 1.0.

There was also no compelling evidence for an effect of mutation position on risk associated with *AR *CAG repeat length. *BRCA1 *mutations were firstly divided by position relative to amino acid 1365 (nucleotide 4213), because mutations 5' of this have been reported to exhibit markedly decreased AR transactivation ability [11]. The RR (95% CI) for the ≥ 28 CAG cut-point analyses were 0.93 (0.53–1.64) for domain 1 (*P *= 0.8) and 0.35 (0.12–1.02) for domain 2 (*P *= 0.06), with marginal evidence for an interaction (*P *= 0.1). *BRCA1 *mutations were also divided by position relative to amino acid 1065 (nucleotide 3311), because the 3' end of the *BRCA1 *fragment has been shown *in vitro *to bind the *AR *amino-terminal domain containing the CAG-encoded polyglutamine tract [11]. The RR (95% CI) for the ≥ 28 CAG cut-point analyses were 0.98 (0.54–1.78) for domain 1 (*P *= 0.9) and 0.45 (0.16–1.23) for domain 2 (*P *= 0.1), with no evidence for an interaction (*P *= 0.3)

There was no convincing rationale for stratification of the *BRCA2 *mutation carriers by mutation position, in that there is no published information available with regard to the domains of *BRCA2 *required for BRCA2–AR interaction or to the effect of *BRCA2 *mutation position on the BRCA2–AR interaction. However, because a single report has shown that the *BRCA2 *L1042X mutation does not enhance AR transactivation [10], mutations 5' to this mutation site might be expected to have similar drastic consequences, and to convey increased risk among carriers with large *AR *CAG alleles. Stratification by mutation position relative to amino acid 1042 (nucleotide 4213) divided the *BRCA2 *carrier sample into two subgroups of 42 and 177 individuals. None of the 42 individuals with mutations in domain 1 had alleles with repeat length ≥ 28 CAG, precluding estimates of risk for this subgroup. However, there was no evidence for increased risk among *BRCA2 *carriers with mutations in the domain 5' to amino acid L1024, with a RR (95% CI) for the ≥ 28 CAG cut-point of 1.11 (0.54–2.30) for the 5' domain (*P *= 0.8).

## Discussion

Our study found no evidence to support the previously reported association of *AR *allele length with increased breast cancer risk in *BRCA1 *carriers [7]. The hypothesis-generating study estimated a 1.8-fold risk (95% CI 1.1–3.1) in *BRCA1 *carriers with at least one *AR *allele length ≥ 28 CAG repeats, and increasing risks of 2.7-fold and 4.5-fold for the ≥ 29 CAG repeat and ≥ 30 CAG repeat cut-points, respectively [7]. The RR estimated by our study for ≥ 28 repeats was 0.74, and the upper 95% confidence limit (1.28) excludes the effect size reported by Rebbeck and coworkers [7]. Moreover, there was no evidence for increased RRs at the ≥ 29 or ≥ 30 cut-points. Given that we had approximately 80% or more power to detect risk estimates previously reported for the ≥ 29 cut-point [7], these results suggest that if there is any increased risk for large number of CAG repeats among *BRCA1 *mutation carriers, then it is of much lower magnitude than was first reported. Rebbeck and coworkers [7] found no effect of *AR *CAG repeat length when considered as a continuous variable, and our study likewise found no evidence for an association with shorter, larger, or average CAG repeat length.

Other published studies have found no evidence to support an association between *AR *CAG repeat length and breast cancer risk in *BRCA2 *mutation carriers [8,9]. Our study also found no evidence in support of an association. Although our sample of 220 *BRCA2 *carriers was not sufficiently large to provide precise estimates of risk, the upper 95% confidence limits imply that a 2.5-fold risk associated with ≥ 28 or ≥ 29 repeat lengths is unlikely.

## Conclusion

Our analyses provide no support for an association between *AR *CAG repeat length and breast cancer risk in either *BRCA1 *or *BRCA2 *mutation carriers. An increased risk associated with ≥ 28 or ≥ 29 repeat lengths is not compatible with our data. Weak associations cannot be excluded, but analyses involving much larger numbers of carriers would be required to evaluate this possibility.

## Abbreviations

AR = androgen receptor; CI = confidence interval; RR = rate ratio.

## Competing interests

The author(s) declare that they have no competing interests.

## Authors' contributions

ABS was responsible for supervising genotyping design and laboratory work, for data cleaning and analysis, and leading the manuscript preparation. ACA, DLD, NP, DMP and BN assisted in the analytical design and analysis of this study. XC and LK were responsible for assay design and optimization, laboratory work and genotype data cleaning. SP and MRC were responsible for the coordination of the EMBRACE study. PLS assisted with the data management and analysis of this study. GSD, CA and MCS assisted with management and provision of data and DNA from the ABCFS and AJBCS. JLH and GGG initiated the ABCFS, and have been instrumental in the ongoing execution of this study. GC-T initiated and obtained funding for this study, initiated collaborative efforts and was involved in the supervision of laboratory work. DFE initiated the EMBRACE study, and assisted in the development of the analytical design of this study and the manuscript preparation. All authors reviewed the manuscript and offered comments.

## Figures and Tables

**Table 1:** Characteristics of study subjects

Sample sources	*BRCA1*		*BRCA2*		*BRCA1 *and *BRCA2*^a^
	
	*n*	(% of total)	*n*	(% of total)	*n*
EMBRACE	247	(64)	92	(42)	0
kConFaB	96	(25)	84	(38)	0
AJBCS	19	(5)	22	(10)	0
ABCFS	20	(5)	23	(10)	1
**Total**	**382**		**221**		1
					
Affected breast cancer^b^	205	(54)	125	(57)	1
Affected ovarian cancer^b^	24	(6)	8	(4)	0
					
Number of families	257		118		1

Questionnaire information on potential confounders was available for 344 *BRCA1 *carriers (239 EMBRACE, 80 kConFaB, 10 AJBCS and 15 ABCFS) and 200 *BRCA2 *carriers (92 EMBRACE, 76 kConFaB, 12 AJBCS, and 20 ABCFS). ^a^One individual was found to carry a deleterious mutation in both *BRCA1 *and *BRCA2 *[19]. ^b^Cancer type refers to first primary cancer diagnosis. One *BRCA2 *carrier with breast cancer was censored as unaffected at age of prior mastectomy. ABCFS, Australian Breast Cancer Family Study; AJBCS, Australian Jewish Breast Cancer study; EMBRACE, Evaluation of Mutant BRCA Carrier Epidemiology study; kConFaB, Kathleen Cuningham Consortium for Research into Familial Breast Cancer.

**Table 2:** Risk associated with *AR *CAG repeat length amongst *BRCA1 *mutation carriers

Risk allele	Adjusted group, ethnicity		Adjusted group, ethnicity and additional variables	
	
	*P*	RR (95% CI)	*P*	RR (95% CI)
*AR *≥ 28 CAG	0.3	0.74 (0.42–1.29)	0.6	0.88 (0.53–1.46)
*AR *≥ 29 CAG	0.3	0.76 (0.43–1.33)	0.7	0.89 (0.54–1.48)
*AR *average CAG	0.5	1.02 (0.96–1.09)	0.3	1.03 (0.97–1.10)
*AR *small CAG	0.3	1.03 (0.97–1.09)	0.2	1.04 (0.98–1.10)
*AR *large CAG	0.9	1.01 (0.96–1.06)	0.6	1.01 (0.97–1.06)

First primary breast cancer diagnosis was considered an event (status affected), whereas first primary ovarian cancers were censored as unaffected at age of diagnosis, and individuals without breast or ovarian cancer were censored as unaffected at age at interview. All individuals were censored as unaffected at age of prophylactic mastectomy prior to diagnosis/interview. Analyses were adjusted for source group, ethnicity, year of birth and hormonal variables oophorectomy, parity, age at menarche and contraceptive pill use. Oophorectomy and parity were treated as time-dependent variables from age at first variable event. Analyses were conducted using unweighted Cox regression.

**Table 3:** Risk associated with *AR *CAG repeat length amongst *BRCA2 *mutation carriers

Risk allele	Adjusted group, ethnicity		Adjusted group, ethnicity and additional variables	
	
	*P*	RR (95% CI)	*P*	RR (95% CI)
AR ≥ 28 CAG	0.8	1.12 (0.55–2.25)	0.9	1.04 (0.47–2.32)
AR ≥ 29 CAG	0.8	0.88 (0.37–2.09)	0.8	0.90 (0.32–2.52)
AR average CAG	0.7	0.98 (0.91–1.06)	1.0	1.00 (0.92–1.09)
AR small CAG	0.6	0.98 (0.90–1.06)	1.0	1.00 (0.92–1.09)
AR large CAG	0.9	0.99 (0.93–1.06)	1.0	1.00 (0.93–1.07)

First primary breast cancer diagnosis was considered an event (status affected), whereas first primary ovarian cancers were censored as unaffected at age of diagnosis, and individuals without breast or ovarian cancer were censored as unaffected at age at interview. All individuals were censored as unaffected at age of prophylactic mastectomy before diagnosis/interview. Analyses were adjusted for source group, ethnicity, year of birth, and hormonal variables oophorectomy, parity, age at menarche, and contraceptive pill use. Oophorectomy and parity were treated as time-dependent variables from age at first variable event. Analyses were conducted using unweighted Cox regression.
